# Evidence for Succession and Putative Metabolic Roles of Fungi and Bacteria in the Farming Mutualism of the Ambrosia Beetle Xyleborus affinis

**DOI:** 10.1128/mSystems.00541-20

**Published:** 2020-09-15

**Authors:** L. A. Ibarra-Juarez, M. A. J. Burton, P. H. W. Biedermann, L. Cruz, D. Desgarennes, E. Ibarra-Laclette, A. Latorre, A. Alonso-Sánchez, E. Villafan, G. Hanako-Rosas, L. López, M. Vázquez-Rosas-Landa, G. Carrion, D. Carrillo, A. Moya, A. Lamelas

**Affiliations:** a Red de Estudios Moleculares Avanzados, Instituto de Ecología A. C., Xalapa, México; b Chair of Forest Entomology and Protection, University of Freiburg, Freiburg, Germany; c Tropical Research and Education Center, University of Florida, Homestead, Florida, USA; d Red de Biodiversidad y Sistemática, Instituto de Ecología A. C., Xalapa, México; e Institute for Integrative Systems Biology (Universitat de València and CSIC), València, Spain; f Foundation for the Promotion of Sanitary and Biomedical Research in the Valencian Community (FISABIO), València, Spain; University of Connecticut

**Keywords:** microbiome, mycobiome, *Xyleborus affinis*

## Abstract

The bacterial and fungal community involved in ambrosia beetle fungiculture remains poorly studied compared to the famous fungus-farming ants and termites. Here we studied microbial community dynamics of laboratory nests, adults, and brood during the life cycle of the sugarcane shot hole borer, Xyleborus affinis. We identified a total of 40 fungal and 428 bacterial operational taxonomic units (OTUs), from which only five fungi (a *Raffaelea* fungus and four ascomycete yeasts) and four bacterial genera (*Stenotrophomonas*, *Enterobacter*, *Burkholderia*, and *Ochrobactrum*) can be considered the core community playing the most relevant symbiotic role. Both the fungal and bacterial populations varied significantly during the beetle’s life cycle. While the ascomycete yeasts were the main colonizers of the gallery early on, the *Raffaelea* and other filamentous fungi appeared after day 10, at the time when larval hatching happened. Regarding bacteria, *Stenotrophomonas* and *Enterobacter* dominated overall but decreased in foundresses and brood with age. Finally, inferred analyses of the putative metabolic capabilities of the bacterial microbiome revealed that they are involved in (i) degradation of fungal and plant polymers, (ii) fixation of atmospheric nitrogen, and (iii) essential amino acid, cofactor, and vitamin provisioning. Overall, our results suggest that yeasts and bacteria are more strongly involved in supporting the beetle-fungus farming symbiosis than previously thought.

**IMPORTANCE** Ambrosia beetles farm their own food fungi within tunnel systems in wood and are among the three insect lineages performing agriculture (the others are fungus-farming ants and termites). In ambrosia beetles, primary ambrosia fungus cultivars have been regarded essential, whereas other microbes have been more or less ignored. Our KEGG analyses suggest so far unknown roles of yeasts and bacterial symbionts, by preparing the tunnel walls for the primary ambrosia fungi. This preparation includes enzymatic degradation of wood, essential amino acid production, and nitrogen fixation. The latter is especially exciting because if it turns out to be present *in vivo* in ambrosia beetles, all farming animals (including humans) are dependent on atmospheric nitrogen fertilization of their crops. As previous internal transcribed spacer (ITS) metabarcoding approaches failed on covering the primary ambrosia fungi, our 18S metabarcoding approach can also serve as a template for future studies on the ambrosia beetle-fungus symbiosis.

## Abstract (precis)

Ambrosia beetles farm their own food fungi within tunnel systems in wood and are among the three insect lineages performing agriculture (the others are fungus-farming ants and termites). In ambrosia beetles, primary ambrosia fungus cultivars have been regarded essential, whereas other microbes have been more or less ignored. Our KEGG analyses suggest so far unknown roles of yeasts and bacterial symbionts, by preparing the tunnel walls for the primary ambrosia fungi. This preparation includes enzymatic degradation of wood, essential amino acid production, and nitrogen fixation. The latter is especially exciting because if it turns out to be present *in vivo* in ambrosia beetles, all farming animals (including humans) are dependent on atmospheric nitrogen fertilization of their crops. As previous internal transcribed spacer (ITS) metabarcoding approaches failed on covering the primary ambrosia fungi, our 18S metabarcoding approach can also serve as a template for future studies on the ambrosia beetle-fungus symbiosis.

## INTRODUCTION

Many arthropods, especially insects and mites, engage in symbiotic mutualisms with fungi (1). Only three insect lineages have convergently evolved to farm fungi for nutritional purposes (i.e., advanced fungiculture): fungus-farming termites, attine ants, and ambrosia beetles (1, 2). Farming involves the selection of beneficial fungi over less beneficial (or antagonistic) fungi, a task that is more easily managed by groups of individuals exhibiting division of labor (3). For ants and termites, it has been shown that bacteria play a prominent role in the farming practices (4), in particular by defending the fungal crops against pathogens (5, 6) but also by nitrogen fertilization of fungus cultivars (7, 8) as well as the enzymatic degradation of plant biomass (9, 10) and plant defenses (11).

Ambrosia beetles (Curculionidae: Scolytinae and Platypodinae) are a polyphyletic group of at least 11 independently evolved wood-boring weevil lineages and are defined by an obligate nutritional dependency on fungi (“ambrosia fungi”), farmed in self-bored tunnels within the xylem of trees (12). The most species-rich lineage of ambrosia beetles belong to the scolytine tribe Xyleborini, with several thousand species (12). Species in this lineage are all haplodiploid, and mating is almost exclusively through inbreeding in the natal nest. Some Xyleborini species are among the most advanced fungus farmers, reflected by cooperative breeding found in these species (13, 14). Cooperative breeding in Xyleborini is characterized by division of labor between mothers (= nest foundresses), adult females, and larval offspring. Adults engage in nest protection and brood and fungus care, whereas larvae take over nest cleaning and expansion (13, 15, 16). Many of these behaviors might involve the application of “bacterial helpers” that might fertilize gardens (e.g., by nitrogen fixation), assist the fungal crops with detoxification of plant defensive compounds and degradation of plant cell walls (e.g., by enzyme production) or defense against pathogens (e.g., by antibiotic production). All these functions have been found to be undertaken by bacterial symbionts in related, phloem-feeding bark beetles (4, 17–21).

Ambrosia beetles bore tunnel systems (= galleries) in the xylem of unhealthy or recently dead trees. On their tunnel walls, they cultivate monocultures of mutualistic ambrosia fungi that grow among a background microbiota of other filamentous fungi, yeasts, and bacteria. Visually (both macro- and microscopically), ambrosia fungi dominate in occupied and active nests and the beetles seem to pick out unwanted fungi and keep the ambrosia cultures pure (22–25). All the other microorganisms in the background are more subtle and almost always ignored by researchers except to mention that they “take over” when the farms are abandoned (13, 15, 23). Whereas ambrosia fungi are transmitted from the natal nest to new nests by nest foundresses in specialized spore-carrying organs, termed mycetangia, and only rarely within the gut (26–28), other microbial associates are found only rarely in mycetangia but are instead transmitted in the gut or on the beetle’s surface. Environmental acquisition from the substrate is also possible (but not in the laboratory assay used in this study). The roles of the symbionts are poorly understood, but symbiont communities certainly comprise beetle mutualists and antagonists. Proven nutritional mutualists are the so-called ambrosia fungi in the ascomycete orders Ophiostomatales (e.g., genus *Raffaelea*), Microascales (e.g., genus *Ambrosiella*), and less frequently, Hypocreales (1, 12, 25). Each genus of ambrosia fungi is typically associated with a specific lineage of beetles and a specific type of mycetangia (29–31). Within these lineages, some beetle species can exchange their primary ambrosia fungi (32, 33), whereas others do appear to have species-specific mutualisms (e.g., reference 29). Saccharomycete yeasts have been found in species-specific relationships with scolytine ambrosia beetles of the genus *Xyleborus* (including our study species X. affinis), a genus that often shows only unspecific relationships with *Raffaelea* ambrosia fungi (32). Therefore, a coevolved mutualistic or parasitic role for these yeasts is possible in *Xyleborus* ambrosia beetles but currently unproven.

Previous studies have indicated that fungal communities of ambrosia beetles are dynamic, both spatially within their galleries and temporally throughout development with the relative abundances of the different symbionts changing over time (34–38). A succession of different fungal (and bacterial) species should be expected, because as the gallery matures, the surrounding wood substrate dries out and is probably degraded by fungal enzymes (39). Larvae may depend on different symbionts than adults, and control over symbionts in their nests and within their bodies (i.e., mycetangium) is particularly important for nest-founding females just prior to emergence, as they need to transmit the beneficial “starter cultures” from the natal nest. Whether the beetles are able to influence symbiont communities and their succession within galleries is unknown, but some evidence suggests that they can. Both larvae and adults have been shown to (i) hinder the spread of experimentally introduced fungal antagonists (13, 15) and (ii) promote the growth of fungal mutualists (40, 41). The mechanisms by which beetles are able to affect fungal growth are still unknown, but they may involve mechanical removal, oral secretions, and the application of mutualistic bacteria (15, 20, 42). Symbiont communities within their bodies and particularly mycetangial symbionts can certainly be influenced by the beetles, as adult females prior to dispersal are known to activate their mycetangia, which are more or less selective for particular ambrosia fungus taxa (43, 44).

There is a long history of studying fungal and bacterial associates in ambrosia beetles, e.g., by using traditional culturing techniques (32, 34, 36–38, 45) or culture-independent approaches (4, 27, 46, 47). Some of these studies have monitored fungal symbionts over time (37, 38, 48–50). Nevertheless, no study has monitored the fungal and bacterial communities associated with all the offspring’s life stages and throughout the development of a beetle gallery using metabarcoding. This kind of study has revealed roles of specific and apparently essential symbionts in leaf-cutter ants, for example (11). In ambrosia beetles, dynamics of symbionts are poorly known, and therefore, we can currently only make guesses about the roles of specific symbionts and their interactions in the development of the beetles.

**FIG 1:**
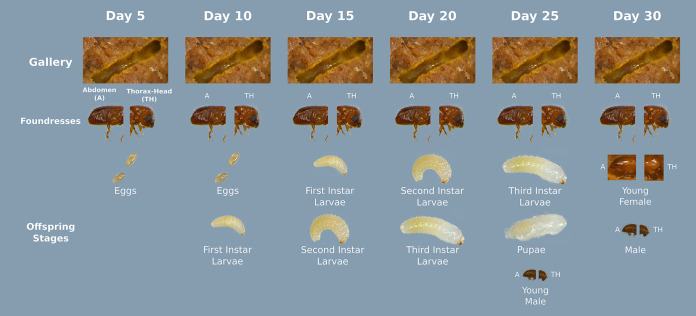
Sample collection and experimental procedure. Six colonies were dissected every 5 days. Samples from the galleries, foundresses, and all offspring at the life stages available at specific time points were collected. The abdomen (A) and thorax-head (TH) were processed independently.

Here we report the first fungal and bacterial metabarcoding study of the symbiont communities of galleries, gallery foundresses, and offspring of all life stages in the sugarcane shot hole borer, Xyleborus affinis Eichhoff. Despite its abundance and pest status (51, 52), microbial symbionts of X. affinis remain poorly studied, and the main ambrosia-fungus mutualist(s) have not been determined for this species, even though several candidates like Raffaelea arxii, a *Candida* sp., and an *Ambrosiozyma* sp. have been discussed as potential mutualists (32). We used a laboratory rearing technique (53) that allows tracking the development of the beetles and collecting samples of specimen and galleries at specific time points, from particular life stages and from the beetle’s oral mycetangia (Fig. 1). Finally, we predict metabolic functions of the microbial communities by using public metabolic databases.

## RESULTS

After quality and chimeric sequence filtering, 5,084,456 bacterial reads and 8,009,205 fungal reads were obtained, with a mean (± standard deviation [SD]) of 181,587 ± 229,231 bacterial and 29,970 ± 21,813 fungal reads per sample (see Table S1 at https://doi.org/10.6084/m9.figshare.12477593). The reads were grouped into 428 bacterial operational taxonomic units (OTUs) (97% homology) and 40 fungal OTUs (99% homology).

The 428 bacterial OTUs belonged to eight different phyla (*Acidobacteria* [0.23%], *Actinobacteria* [8.88%], *Bacteroidetes* [10.51%], *Chloroflexi* [0.23%], *Firmicutes* [13.79%], *Proteobacteria* [64.49%], *Tenericutes* [0.23%], and TM7 [0.93%]) (Fig. S1). Bacterial taxonomic cladograms differed between the types of the samples (galleries, foundresses, offspring) (Fig. S1). While *Gammaproteobacteria* (*Enterobacter* and *Stenotrophomonas*) predominated in galleries, *Betaproteobacteria* (*Burkholderiaceae*, *Alcaligenaceae*, and *Comamonadaceae*), *Alphaproteobacteria* (*Sphingomonadaceae* and *Brucellaceae*), *Sphingobacteria* (*Sphingobacterium*), *Bacteroidetes* (*Chryseobacterium*), and *Actinobacteria* (*Mycobacteriaceae*, *Gordoniacea*, and *Tsukamurellaceae*) made up the bacteriome of foundresses and offspring, with *Actinobacteria* being the most abundant and diverse in foundresses (Fig. S1).

10.1128/mSystems.00541-20.1 FIG S1 Taxonomic cladogram of the bacterial OTUs obtained by 16S metabarcoding. (A) All combined samples, (B) offspring life stages combined (eggs, larvae, pupae, and nonsclerotized offspring), (C) galleries, (D) foundresses. Nodes represent the taxonomic classification kingdom, phylum, class, order, family, and genus, from inside out. The labels on the rings indicate genera and families. The sizes of the nodes are representative of their relative abundances. Download FIG S1, TIF file, 0.8 MB. Copyright © 2020 Ibarra-Juarez et al. 2020 Ibarra-Juarez et al. This content is distributed under the terms of the Creative Commons Attribution 4.0 International license.

The fungal OTUs were classified in six orders (Mucorales [5%], Hypocreales [10%], Saccharomycetales [32.5%], Microascales [5%], Ophiostomatales [15%], and Eurotiales [2.5%]) (Fig. S2). The relative abundance of the different taxa in the fungal microbiome also varied between the types of samples (galleries, foundresses, and offspring). While only a few taxa in the Saccharomycetales (*Candida* and *Saccharomycopsis*), Ophiostomatales (*Raffaelea*), Eurotiales (*Talaromyces*), Hypocreales (*Fusarium*), and Mucorales predominated in the galleries, additional Saccharomycetales (plus *Cyberlindnera* and *Meyerozyma*) and Microascales (*Graphium*) dominated in the foundresses and offspring (Fig. S2). Relative abundance measures based on 16S/18S amplicon sequencing analyses have to be treated with care, however, because relative abundances based on ribosomal genes do not directly translate to physical abundance of the specific microbes (54). Semiquantitative comparisons between samples are possible, however.

10.1128/mSystems.00541-20.2 FIG S2 Taxonomic cladogram of the fungal OTUs obtained by 18S metabarcoding. (A) All combined samples, (B) foundresses, (C) galleries, (D) offspring life stages combined (larvae, pupae, and nonsclerotized offspring). Nodes represent the taxonomic classification kingdom, phylum, class, order, family, and genus, from inside out. The labels on the rings indicate genera and families. The size of the nodes is representative of their relative abundances. Download FIG S2, TIF file, 0.4 MB. Copyright © 2020 Ibarra-Juarez et al. 2020 Ibarra-Juarez et al. This content is distributed under the terms of the Creative Commons Attribution 4.0 International license.

Overall, both fungal and bacterial OTU richness and diversity varied between samples and throughout beetle development (for details, see Fig. S3 to S8 at https://doi.org/10.6084/m9.figshare.12477593).

### Structure of fungal and bacterial communities throughout beetle development.

**FIG 2:**
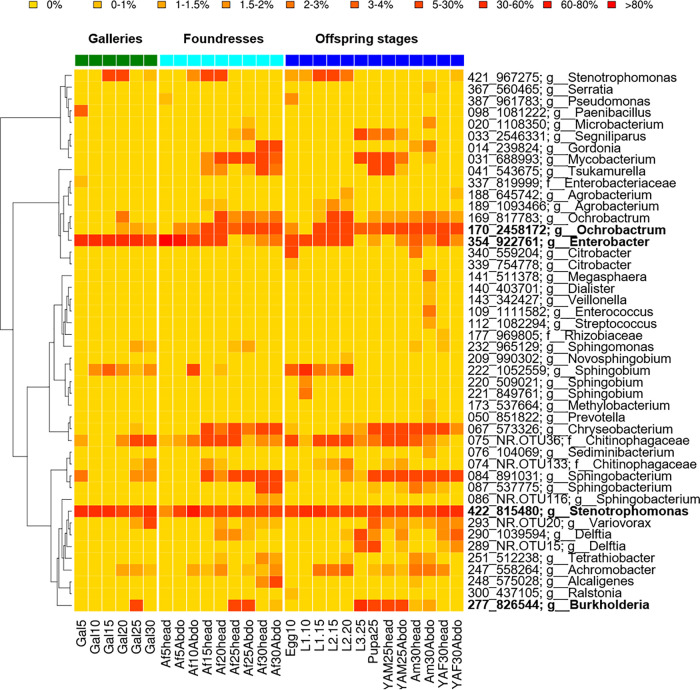
Relative abundances of the 46 most abundant (>0.1%) bacterial OTUs per sample within galleries, foundresses, and offspring of different life stages of *X. affinis*. The predominant bacterial symbionts (abundance of >20% across samples), an *Ochrobactrum* OTU, an *Enterobacter* OTU, a *Stenotrophomonas* OTU, and a *Burkholderia* OTU, are given in bold type. Six samples from each gallery (Gal), foundress (adult female [Af]), and offspring life stage (adult male [Am], teneral female [young adult female {YAF}], teneral male [young adult male {YAM}], pupa, larva first to third instar [L1 to L3], egg; mycetangium [head], abdomen [Abdo]) were pooled and collected between 5 and 30 days after gallery foundation. The dendrogram on the left side shows the 16S phylogenetic relationship between the OTUs.

**FIG 3:**
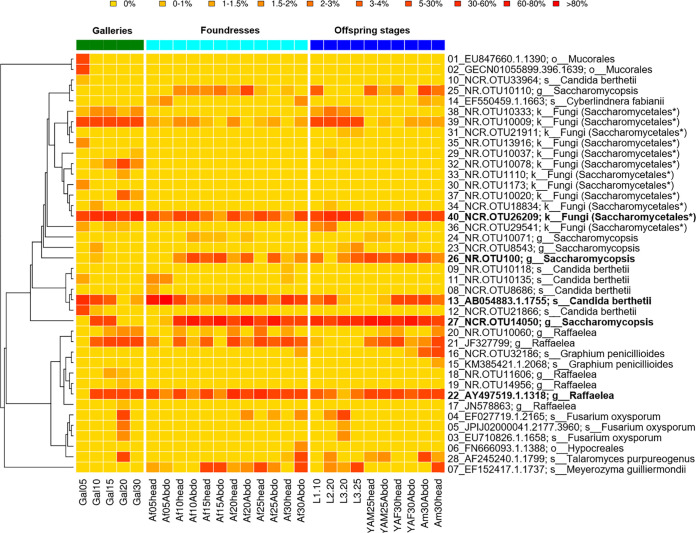
Relative abundance of fungal OTUs per sample within galleries, foundresses, and offspring of the different life stages of *X. affinis*. The predominant fungal symbionts (abundance of >20% across samples), a *Raffaelea* OTU and four OTUs of ascomycete yeasts, are given in bold type. Six samples from each gallery (Gal), foundress (adult female [Af]) and offspring life stages (adult male [Am], teneral female [YAF], teneral male [YAM], larva first to third instar [L1 to L3]; mycetangium [head], abdomen [Abdo]) were pooled and collected between 5 and 30 days after gallery foundation. The dendrogram on the left side shows the 18S phylogenetic relationship between the OTUs. An asterisk shows that these OTUs could not be classified and were automatically assigned to “fungi,” but given their phylogenetic placement, we assigned them to Saccharomycetales.

The abundance of bacterial and fungal OTUs was strongly biased toward certain taxa (Fig. 2 and 3). Forty-six bacterial OTUs had an abundance of >1% (Fig. 2). Only five of these OTUs were relatively abundant in all the samples: a *Stenotrophomonas* (OTU 815480; mean ± SD of 23.9% ± 16.1%), an *Enterobacter* (OTU 922761; 6.42% ± 5.8%), an *Ochrobacter* (OTU 2458172; 5.21% ± 4.12%), a *Chryseobacterium* (OTU 573326; 4.07% ± 4.92%) and a *Sphingobacterium* (OTU 891031; 4.07% ± 4.97%). The two most abundant OTUs, an *Enterobacter* and a *Stenotrophomonas*, dominated communities of galleries (37.4% ± 15.9% and 30.8% ± 9.2%; see Fig. S9 at https://doi.org/10.6084/m9.figshare.12477593), heads (22.11% ± 36.93% and 9.76% ± 6.31%), and abdomens of foundresses (20.74% ± 32.48% and 26.9% ± 23.77%; see Fig. S10 at the figshare URL above), as well as eggs (29.24% and 20.16%) and first/second larval instars (12.49 ± 1.98% and 28.50 ± 11.45%) (see Fig. S11 at the figshare URL above). Both, but in particular *Enterobacter*, changed in frequency along with the beetle’s life stages. They were rare in third instar larvae, pupae, and young males, in which a *Burkholderia* (OTU 826544; 23.99% ± 15.94%) and a *Mycobacterium* (OTU 688993; 10.07 ± 6.15%) OTU predominated. *Enterobacter* was especially abundant within eggs, larvae, and foundresses from young nests.

The relative abundances of the fungal OTUs varied along with the development of galleries and beetle life stages (Fig. 3). Three yeasts (NCR.OTU26209, NCR.OTU14050, and AB054883.1.1755) and two *Raffaelea* OTUs (GenBank accession no. AY497519.1.1318 and JF327799) were widely distributed throughout gallery and beetle samples. Along with gallery development, a *Candida* OTU (AB054883.1.1755) started with a relative abundance of 52.2% at day 5 and decreased down to 1.6% by day 30 (see Fig. S12 at https://doi.org/10.6084/m9.figshare.12477593). In contrast, a *Raffaelea* OTU (AY497519.1.1318) was absent in galleries at day 5, reached 19.5% at day 10, and then kept an abundance of around 40%. An unknown Saccharomycetales yeast (NCR.OTU26209) was abundant throughout gallery development (30.82% ± 11.37%) and may serve as larval food (see below).

A *Raffaelea* OTU (AY497519.1.1318) growing abundantly in galleries was also commonly found in foundresses (15.31% ± 12.45%) and mostly in their heads (22.89% ± 12.92%), suggesting that it colonizes the oral mycetangia (see Fig. S13 at https://doi.org/10.6084/m9.figshare.12477593). In contrast, larvae mostly lacked this *Raffaelea* OTU (3.10% ± 1.40%) but instead contained a *Saccharomycopsis* OTU (NCR.OTU14050; 36.17% ± 19.10%) and the unknown Saccharomycetales yeast (NCR.OTU26209; 32.14% ± 12.20%) (see Fig. S14 at the figshare URL above). In addition to the *Raffaelea* OTU (AY497519.1.1318), the heads and abdomens of foundresses were dominated by two yeasts, a *Candida* OTU (AB054883.1.1755) and the larval *Saccharomycopsis* OTU (NCR.OTU14050), both of which relative dominances within foundress samples fluctuated over time (see Fig. S13 at the figshare URL above).

### Microscopic analyses.

**FIG 4:**
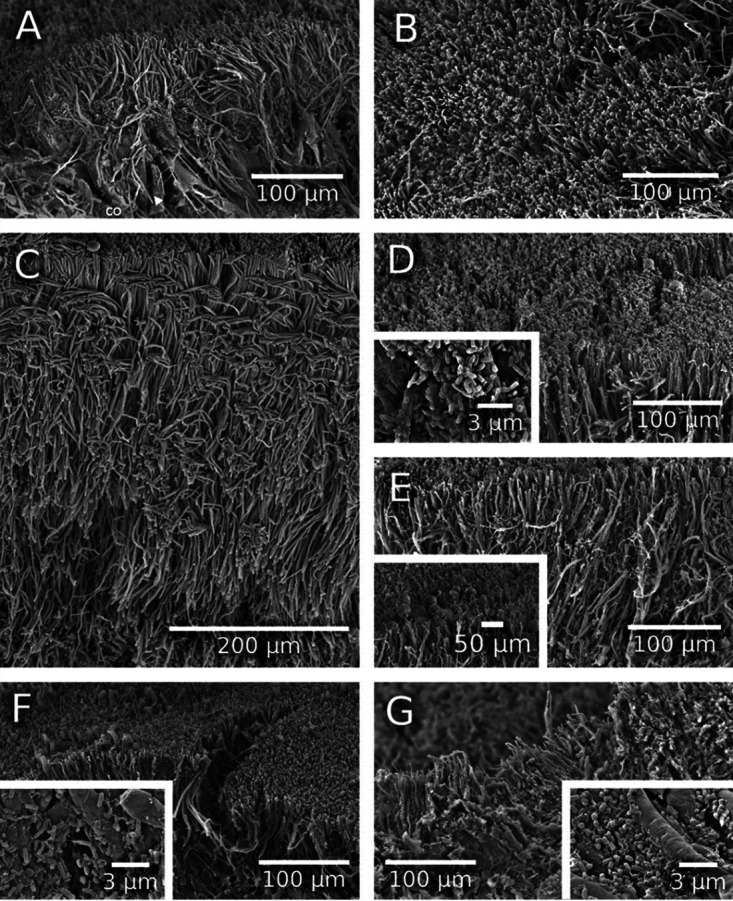
Scanning electron microscopy images of *X. affinis* galleries (1,000× magnification, lateral view). (A) Hyphae and yeast-like cells (possibly *Candida*) on day 3, (B to G) mycelium from yeast and ambrosia fungi on (B) day 5, (C) day 10, detached conidium (co), (D) day 15 (bacteria cells, 30,000× magnification), (E) day 20 (conidia and conidiophores, 2,500× magnification), (F) day 25 (bacteria cells, 30,000× magnification), and (G) day 30 (bacteria cells, 30,000× magnification). See also Fig. S15, S16, and S17 at https://doi.org/10.6084/m9.figshare.12477593.

**FIG 5:**
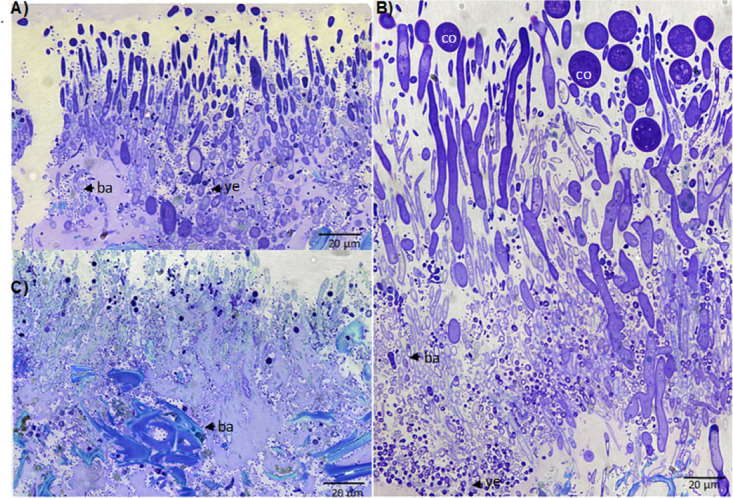
Light microscopy images of transversal sections of tunnel walls through gallery development. (A) Day 5, (B) day 10, (C) day 15. Bacteria (ba), yeast cells (ye), and conidia (co) (possibly *Raffaelea*) are indicated. Magnification, 40×.

Dynamics of the microbial communities were also visualized using gallery samples for scanning electron microscopy (SEM) and light microscopy (LM) (Fig. 4 and 5; see Fig. S15 to S17 at https://doi.org/10.6084/m9.figshare.12477593). Shortly, after gallery foundation, between days 5 and 10, both SEM and LM revealed the establishment of filamentous yeast and bacterial communities on gallery walls (Fig. 4A to C and Fig. 5A and B; see Fig. S15A to E at the figshare URL above). Between days 5 and 10, the yeast mycelium grew from 94.06 (±4.49) μm by SEM and 95.6 (±5.63) μm by LM to its maximum lengths of 379.39 (±10.46) μm by SEM and 221.07 (±6.89) μm by LM. On day 10, a transition from yeast-like to hyphal growth was observed (see Fig. S15F at the figshare URL above), and the first fungal conidiophores and conidia appeared, resembling those of *Raffaelea* and *Fusarium* species (see Fig. S15D and E at the figshare URL above). On day 15, the bacterial abundance increased along with the production of exopolysaccharides (biofilm) (Fig. 4D and 5C; see Fig. S16A at the figshare URL above). Three different bacterial morphotypes could be observed (Fig. 4D, inset). On day 20, *Raffaelea* conidiophores proliferated on the surfaces of the galleries (Fig. 4E; see Fig. S16B and C and Fig. S17A at the figshare URL above). On day 25, only one bacterial morphotype was detected, and the number of *Raffaelea* conidiophores decreased again (Fig. 4F; see Fig. S16D and E, and Fig. S17B at the figshare URL above). On day 30, signs of mycelial degradation and only one bacterial morphotype were seen (Fig. 4G; see Fig. S16F and Fig. S17B at the figshare URL above). Interestingly, transverse sections of galleries (from both SEM and LM; Fig. 4 and 5; see Fig. S17 at the figshare URL above) revealed that the microbial community on gallery walls is composed of three layers: at the bottom are bacteria, and yeasts, then fungal filaments, and on top, *Raffaelea* conidiophores and conidia that can be also seen in the images taken from the top (Fig. 4; see Fig. S16 at the figshare URL above).

### Putative metabolic function of the microbiota over time.

To further investigate the bacterial metabolic shift over time, we predicted the metabolic profile of the samples using PICRUSt software. On the basis of permutational multivariate analysis of variance (PERMANOVA) and nonmetric multidimensional scaling (NMDS) analyses, we found that time (days 5, 10, 15, 20, 25, and 30) and sample type (adult beetles [= foundresses and nonsclerotized offspring], galleries, and immature offspring [= eggs, larvae, and pupae]) determined the metabolic profile of the bacterial community. The PERMANOVA of KEGG functional orthologs (KOs) (*F*_(2, 55)_ = 12.03, *R*^2^ = 0.32931, *P* = 0.001) and of L3 KEGG level (*F*_(2, 55)_ = 5.08, *R*^2^ = 0.20227, *P* = 0.001) showed significant differences among sample types and also among timing of sampling (for KOs, *F*_(7, 55)_ = 5.74, *R^2^* = 0.39233, *P* = 0.001; for L3, *F*_(7_, _55)_ = 4.36, *R*^2^ = 0.4336, *P* = 0.001) (see Fig. S18 and details in the supplemental material posted at https://doi.org/10.6084/m9.figshare.12477593).

**FIG 6:**
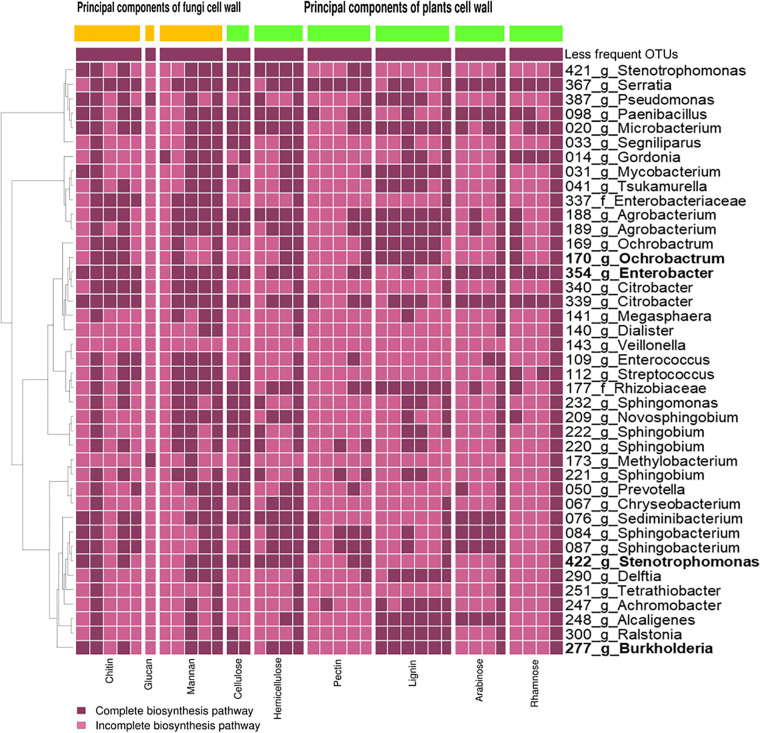
Presence or absence of genes inferred from the KEGG database coding for cell wall-degrading enzymes in the bacterial community found in this study. We display only the OTUs present in the Greengenes database and with a relative frequency of >1% in at least one sample. The predominant bacterial symbionts (abundance of >20% across samples), an *Ochrobactrum* OTU, an *Enterobacter* OTU, a *Stenotrophomonas* OTU, and a *Burkholderia* OTU, are given in bold type. The heatmap shows the presence (dark purple) or absence (light purple) of the catabolic enzymes required for the degradation of a given cell wall component. Every column stands for an individual enzyme required to degrade the respective compound (for details, see Fig. S35 and Table S6 at https://doi.org/10.6084/m9.figshare.12477593). The dendrogram on the left side shows the 16S phylogenetic relationship between the OTUs.

**(i) Degradation of the fungal and plant cell wall.** The bacterial symbionts together can possibly degrade all the major plant and fungal polymers like chitin, glucan, mannan, cellulose, hemicellulose, pectin, lignin, arabinose, and rhamnose (Fig. 6). A few bacterial OTUs can degrade specific compounds on their own: chitin (*Enterobacter* and *Citrobacter*), glucan (*Pseudomonas*), and pectin (*Serratia*). Cellulose, hemicellulose, lignin, arabinose, and rhamnose can be degraded by many OTUs. The four predominating bacterial taxa possibly have the capabilities to fully degrade all polymers except glucan, mannan, and pectin.

**FIG 7:**
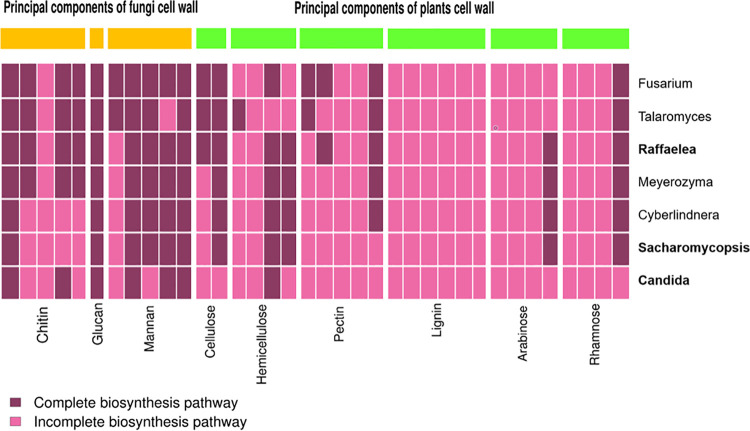
Presence or absence of genes, inferred from fungal genome comparisons, coding for cell wall-degrading enzymes for a subset of the fungal community found in this study. The predominant fungal symbionts (abundance of >20% across samples), a *Raffaelea* strain, a *Saccharomycopsis* strain, and a *Candida* strain, are given in bold type. The heatmap shows the presence (dark purple) or absence (light purple) of the specific enzymes required for the degradation of a given cell wall component. Every column stands for an individual enzyme required to degrade the respective compound (for details, see Fig. S36 and Table S6 at https://doi.org/10.6084/m9.figshare.12477593).

The enzymatic capabilities of the fungal symbionts appear less complete (Fig. 7). Only glucan and mannan can by fully degraded by the joint activity of the fungi. Glucan can be degraded by all fungi, but apart from a *Fusarium* (a relatively uncommon OTU) that possibly has all the genes required for mannan degradation, none of the other symbionts is able to degrade a polymer completely on its own. Among the dominant players, *Raffaelea* is possibly the most potent degrader of plant cells (i.e., cellulose, partly hemicellulose) (Fig. 7).

If putative enzymatic capabilities of OTUs are mapped against sampling time (days 5 to 30) and sample type (adult beetles [= foundresses and nonsclerotized offspring], galleries, and immature offspring [= eggs, larvae, and pupae]), the following pattern appears: cellulose-, hemicellulose-, mannan-, and rhamnose-degrading bacterial symbionts are relatively more abundant during the first half of gallery development (until day 15), whereas symbionts degrading lignin are more abundant during the second half (see Fig. S20 at https://doi.org/10.6084/m9.figshare.12477593). During the second half of gallery development, cellulose, hemicellulose, and rhamnose degradation capabilities are highest on day 30. Cellulose-, hemicellulose-, and mannan-degrading capabilities are more common in symbionts within galleries than in symbionts of adults and immature offspring; the reverse pattern appears for lignin and glucan (see Fig. S21 at the figshare URL above). The dominant genera *Enterobacter*, *Stenotrophomonas*, and *Ochrobactrum* as well as the relatively abundant *Sphingobacterium*, probably play the main role in the degradation of complex sugars (see Fig. S22 and S23 at the figshare URL above).

**FIG 8:**
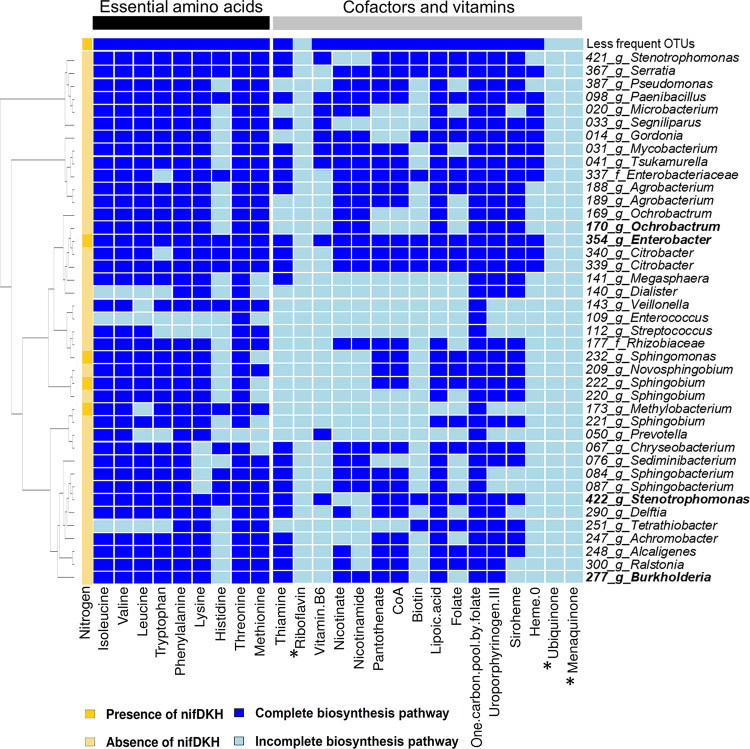
Presence or absence of genes inferred from the KEGG database coding for nitrogen-fixing enzymes, essential amino acids, cofactors, and vitamin biosynthesis pathways in the bacterial community identified in this study. The predominant bacterial symbionts (abundance of >20% across samples), an *Ochrobactrum* OTU, an *Enterobacter* OTU, a *Stenotrophomonas* OTU, and a *Burkholderia* OTU, are given in bold type. The first column shows the presence (dark yellow) or absence (light yellow) of *nifDKH* genes (nitrogenase enzyme complex to fix atmospheric nitrogen). The left heatmap panel shows the amino acid biosynthesis pathways and the right heatmap panel shows cofactors and vitamins biosynthesis pathways (dark blue for complete pathway; light blue for incomplete pathway). Metabolites with an asterisk are not encoded by any bacterial OTU in the microbiome (riboflavin, ubiquinone, and menaquinone). Every column stands for an individual enzyme required to synthesize the molecule. For more details, see Table S6 at https://doi.org/10.6084/m9.figshare.12477593. The dendrogram on the left shows the phylogenetic relationships between the OTUs.

**(ii) Nitrogen fixation and biosynthesis of amino acids, cofactors, and vitamins.** Atmospheric nitrogen fixation is only known from bacteria, not fungi. Among the predominant bacterial symbionts, an *Enterobacter* can possibly fix nitrogen. Additionally, there might be nitrogen fixation by a *Sphingobacterium*, a *Sphingomonas*, and a *Methylobacterium*, but all these were uncommon within the microbiome (Fig. 8). Altogether, these taxa were relatively more abundant in galleries than in adults and immature offspring, and immediately after nest foundation, their abundance decreased (see Fig. S24 and S25 at https://doi.org/10.6084/m9.figshare.12477593).

Regarding essential amino acids, almost all bacterial OTUs can possibly synthesize them except for histidine, which can be only synthetized by 13 OTUs and only two of the most abundant bacterial OTUs (*Enterobacter* and *Stenotrophomonas*) (Fig. 8).

The fungal symbionts altogether are likely not able to synthesize methionine (either EC 2.3.1.46 or EC 2.3.1.31 is absent). Fungal OTUs with capabilities for synthesis of isoleucine, valine, leucine, and threonine increased in abundance along with the progression of the beetle’s life cycle and were generally higher in adults and in immature offspring than in gallery samples (see Fig. S24 and S25 at https://doi.org/10.6084/m9.figshare.12477593). In contrast, OTUs with the capability to produce tryptophan and methionine decreased with development of galleries and were also less common in adults and immature offspring than in gallery samples.

**FIG 9:**
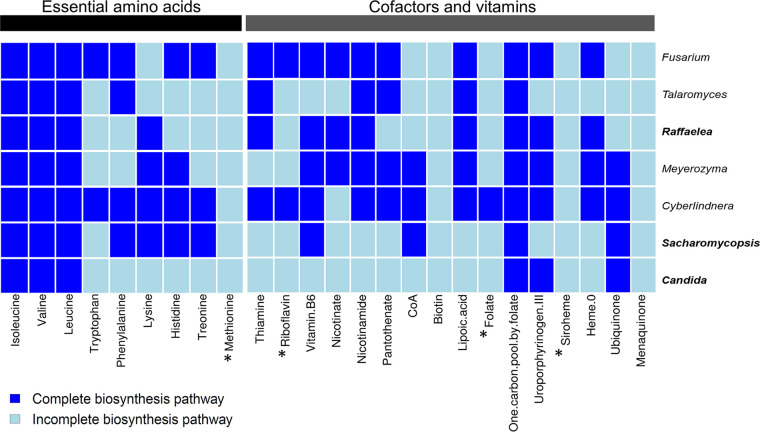
Presence or absence of genes, inferred from fungal genome comparisons, coding for components of biosynthesis pathways of essential amino acids, cofactors, and vitamins in the fungal community identified in this study. The predominant fungal symbionts (abundance of >20% across samples), a *Raffaelea* strain, a *Saccharomycopsis* strain, and a *Candida* strain, are given in bold type. The left heatmap panel shows the amino acid biosynthesis pathways, and the right heatmap panel shows cofactors and vitamins biosynthesis pathways (dark blue for complete pathway; light blue for incomplete pathway). Metabolites with an asterisk lack an enzyme that is not encoded by any fungal OTU in the microbiome (methionine and siroheme). Every column stands for a specific enzyme required to synthesize the molecule. For more details, see Table S6 at https://doi.org/10.6084/m9.figshare.12477593. The dendrogram on the left shows the phylogenetic relationships between the OTUs.

The core fungi *Raffaelea*, *Saccharomycopsis*, and *Candida* possibly lack the genes encoding components needed to synthesize tryptophan, riboflavin, pantothenate, biotin, and folate (Fig. 9). Overall, the nine dominant bacterial and fungal symbionts may jointly synthesize 14 out of the 16 cofactors and vitamins (except riboflavin and menaquinone).

Maximum synthesis of thiamine, vitamin B6, coenzyme A (CoA), biotin, lipoic acid, folate, and the one-carbon pool by folate, siroheme, and heme appears to occur around the first half of gallery development (until day 15) and on day 30, with the exception of siroheme (see Fig. S26 and S27 at https://doi.org/10.6084/m9.figshare.12477593). The biosynthesis of nicotinate and nicotinamide peaked on day 5 and day 30, mainly in galleries for nicotinamide and in adult beetles (foundresses and nonsclerotized offspring) for nicotinate (see Fig. S26 and S27 at the figshare URL above). Pantothenate biosynthesis peaked in adult beetles on day 25. The biosynthesis of uroporphyrinogen III increased as the beetles completed their life cycle, exhibiting a peak on day 30 in the immature offspring samples. *Enterobacter*, *Stenotrophomonas*, *Mycobacterium*, *Ochrobactrum*, and *Sphingobacterium* probably played the main role in the amino acid synthesis (see Fig. S28 and S29 at the figshare URL above). While *Enterobacter* and *Stenotrophomonas* were highly abundant in all the samples, *Mycobacterium* was present only in adult samples (see Fig. S5 at the figshare URL above).

**(iii) Quorum sensing and biofilm production.** The analysis of putative quorum-sensing genes in the bacterial symbionts revealed the presence of four complete systems: Escherichia coli (*luxS/*AI-2 [autoinducer 2]), primarily associated with biofilm production, Xanthomonas campestris (*rpfB*/*rpfF/*DSF [diffusible signal factor]), which is associated with virulence and antibiotic resistance and known to induce Candida albicans hypha formation (55), Enterococcus faecalis (*fsrD/*GBAP [gelatinase biosynthesis-activating pheromone]) that controls the expression of pathogenicity (56), and enterohemorrhagic E. coli (EHEC) (*qseC/*AI-3) known to facilitate the invasion of intestinal epithelia (57) (see Fig. S30 at https://doi.org/10.6084/m9.figshare.12477593). The *luxS*/AI-2 system is probably carried by the dominant *Enterobacter* and a few other more uncommon genera (*Serratia*, *Microbacterium*, *Citrobacter*, *Enterococcus*, *Streptococcus*, and *Prevotella*). Only *Enterobacter* may contain the sensing proteins (see Fig. S30 at the figshare URL above). Its relative frequency decreased as the beetles completed their life cycle (showing a peak on day 5) and was highest within the galleries (see Fig. S31 at the figshare URL above). While the synthesis of DSF (*rpfB/rpfF/*DSF system) could be achieved by 35 out of 39 OTUs, only *Strenothrophomonas* may had the sensing proteins (see Fig. S34 at the figshare URL above). The relative frequency of this OTU increased during the beetles’ life cycle, exhibiting a peak in galleries by day 30 (see Fig. S31 at the figshare URL above). The synthesis of GBAP (*fsrD/*GBAP system) could be accomplished by *Enterococcus*, and the sensing proteins might have been present in *Enterococcus* and *Streptococcus* (see Fig. S30 at the figshare URL above), although both were not part of the core bacterial community. The QseC/AI-3 system showed a peak on day 10 in eggs (see Fig. S31 at the figshare URL above)—possibly as a result of the predominant *Enterobacter*. Overall, analyses of the genes involved in biofilm production indicated that their relative frequencies increased toward the end of the beetles’ life cycle, while the relative frequencies of the genes related to the planktonic stage decreased (see Fig. S32 at the figshare URL above).

## DISCUSSION

**FIG 10:**
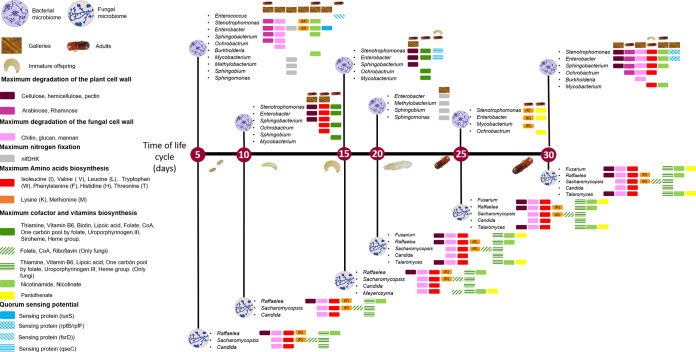
Schematic model of the life cycle of *X. affinis* (center) and the dominant bacterial taxa (top) as well as fungal taxa (bottom) ring the life stages (5-day sampling intervals) and their potential, KEGG- and fungal genome-inferred, metabolic roles. The colored boxes next to the names of the microbes indicate the different metabolic functions that may be carried out by the respective taxa. The symbols for gallery, larvae, and adults above these colored boxes indicate in what sample the respective metabolic functions were probably most prevalent given the microbial OTUs found and their inferred metabolic functions. Multiple boxes of the same color would indicate that a specific function is possibly present in several of the microbes. The lengths of the vertical lines have no specific meaning.

We characterized the fungal and bacterial symbionts associated with *X. affinis* during its life cycle using a metabarcoding approach (for an overview, see Fig. 10). Even though bacteria and yeasts are long known to be common in ambrosia beetle galleries (22, 23), the filamentous fungal symbionts, in particular *Raffaelea* and *Ambrosiella* ambrosia fungi, are regarded as the main source of nutrition for both adults and larvae (1, 25). The role of the rest of the microbial community is relatively unclear, even though positive effects of secondary compounds produced by bacteria or yeasts on bark and ambrosia beetles or their fungal mutualists, respectively, have been found (e.g., references 19, 42, 58, and 59). It has never been investigated in ambrosia beetles, however, whether symbiotic bacteria and yeasts may help to degrade plant polymers, fix atmospheric nitrogen, and supplement the beetles and their primary fungi with other essential amino acids, cofactors, and vitamins. Such essential roles of symbionts have been found in the other fungus-farming systems of ants and termites, however (6–10).

### The bacterial community.

The microbiome of *X. affinis* consisted of a total of 428 bacterial OTUs (100 genera) and 40 fungal OTUs (8 genera) with only 46 bacterial OTUs (35 genera) with a relative abundance of >1%. Out of these OTUs, only four bacterial and five fungal OTUs had a relative abundance of >20%. This small number of the most frequent OTUs agrees with observations on bark beetles (71) and other specialist insects like bees (60) and disagrees with previous studies on ambrosia beetles that found much higher diversities (e.g., on *X. affinis*) (47, 61, 62). However, there are two major problems with the latter studies. First, they used field-collected ambrosia beetles caught in traps, where the specimens sat together for some time in collection liquids that were possibly contaminated with microbes from the environment and other caught insects. Second, their metabarcoding methods were inadequate for the ambrosia beetles that we focus on, because their models are associated with Ophiostomatales fungi for which internal transcribed spacer (ITS) metabarcoding does not work (63–65). In contrast, our 18S metabarcoding approach delivered results that are consistent with a previous isolation study, which has shown a lower abundance of OTUs in lab-reared ambrosia beetles than in wild-reared ambrosia beetles (46).

The most abundant and widely distributed bacterial genera were *Enterobacter* and *Stenotrophomonas.* Both genera have been isolated from other bark and ambrosia beetles (4, 27, 46, 66–70). Their relative abundance negatively correlated with the diversity of bacteria, suggesting that they might be able to structure (i.e., dominate) the bacterial communities, possibly by secondary metabolites (see Fig. 33 at https://doi.org/10.6084/m9.figshare.12477593).

### The fungal community.

Forty fungal OTUs composed the fungal community, with Saccharomycetales and Ophiostomatales (both Ascomycota) as the most abundant orders. Among these OTUs, the five most abundant OTUs belonged to the genera *Saccharomycopsis*, *Candida*, and *Raffaelea*. These results correspond with the culturing results from other bark and ambrosia beetles (25, 38, 46, 71). Both our metabarcoding and SEM visualizations showed that the yeasts are dominant in young galleries, after which filamentous *Raffaelea* species follow. This is a surprising finding, because the filamentous ambrosia fungi (in the *Raffaelea* and *Ambrosiella* genus) are usually regarded as the main food fungi of ambrosia beetles (12, 25). Yeasts are common associates of bark and ambrosia beetles, even though their role is poorly understood (18, 58). A recent study found, however, species-specific associations of yeasts with *Raffaelea*-farming scolytine ambrosia beetles (32), which suggests mutualistic (or parasitic) coevolution. Interestingly, this study reported a Candida berthetii isolate to be specific to our study species, *X. affinis*, which we also determined as one of the most abundant yeasts in our samples (32). In our study, the relative abundance of C. berthetti peaked in the heads of adult female offspring on day 30, when these females are about to leave the nest. This may suggest that this yeast is vertically transmitted to new nests in oral mycetangia of nest foundresses. The presence of *C. berthettii* within the mycetangia of *X. affinis* has also been reported by the culturing study mentioned above (32). All these findings agree with an old hypothesis of yeasts being pioneer colonizers that assist with the preparation of the niche for the growth of the filamentous *Raffaelea* ambrosia fungi (23, 72).

Both our LM and SEM images and the molecular data confirmed that *Raffaelea* fungi appeared within galleries after the hatching of eggs (day 10 versus day 5), which indicates that yeasts are the primary food source at least for the early instars of larvae and possibly also for the egg-laying mother. Furthermore, our molecular data showed that both yeasts were common in the larvae and abdomens of surface-cleaned foundresses, in particular from young nests. This finding is new for scolytine ambrosia beetles, because all previous studies described the filamentous *Raffaelea* or *Ambrosiella* fungi as the principal food source for both larval and adult ambrosia beetles (37, 38, 53, 73). It confirms findings from a culturing study on the platypodine ambrosia beetle, Platypus cylindrus, however, in which galleries a yeast, Endomycopsis platypodis, established before the primary *Raffaelea* fungus (74). The *Raffaelea* fungi probably serve as a food source after 10 to 15 days. Between days 25 and 30, our SEM pictures showed many cropped conidiospores of *Raffaelea*, which might suggest that the reproduction of conidia declines around that time. This also coincides with the time when the preemerging females load their mycetangia with fungal propagules (likely yeasts and *Raffaelea* conidia) that they transmit to newly founded galleries (24, 43, 44). At that time, yeasts and bacteria were found in layers at the bottom below the *Raffaelea* conidiophores (Fig. 5), possibly suggesting some metabolic division of labor in enzymatic degradation of the wood between the different microbes.

### Putative metabolic functions.

Ambrosia beetles obviously farm primary ambrosia fungi such as *Raffaelea* and possibly yeasts (see above), which have been thought to degrade the wood surrounding the beetle galleries (e.g., references 39 and 75). Our KEGG analysis allowed us to predict the metabolic functions of about 60% of the bacterial OTUs. Most importantly, this analysis and the fungal genome comparisons included all the core bacterial and fungal OTUs, so it is likely that our analysis covers the most important metabolic functions. Overall, it suggested that the bacterial microbiome may be able to assist in wood and fungal biomass degradation. This is indicated by the four most abundant bacterial OTUs being jointly capable to fully degrade all the fungal and plant polymers except glucan, mannan, and pectin, whereas the five most abundant fungi can fully degrade only glucan and cellulose and partly degrade hemicellulose (Fig. 6 and 7). Whether the degradation of these polymers by bacteria plays an important nutritional role for the ambrosia beetles needs to be determined in future studies.

The bacterial genera *Enterobacter* and *Stenotrophomonas* are particularly abundant during the first days of gallery development, followed by the yeasts and the *Raffaelea* ambrosia fungi. We hypothesize that plant cell wall degradation is carried out initially by the bacterial and yeast microbiome, supplementing carbohydrates for the *Raffaelea* fungi. After day 10, this function is performed jointly by the filamentous fungi, yeasts, and bacteria. The latter are found particularly along the woody surface of the wall, at the bottom of the *Raffaelea* fungus layer that covers the gallery walls (Fig. 5). The breakdown of complex sugars like cellulose, hemicellulose, and pectin first peaked around days 10 to 15, which coincides with a high energy demand of the growing fungi and a strong increase in the number of larvae. A second peak of sugar breakdown was observed around day 30, coinciding with the emergence of the F1 generation. Energy demands around that time are probably very high because preemerging adult female offspring fill their mycetangia (43, 75), build up fat reserves for dispersal (76), and produce juvenile hormone (77). The bacterial genera *Enterobacter* and *Stenotrophomonas* are particularly abundant during the first days of gallery development, followed by the yeasts and *Raffaelea* ambrosia fungi. We hypothesize that plant cell wall degradation is carried out initially by the bacterial and yeast microbiome, supplementing carbohydrates for the *Raffaelea* fungi. After day 10, this function is performed jointly by the fungal and bacterial microbiomes. The highest KO degradation relative frequency picks of the complex sugars (cellulose, hemicellulose, and pectin) took place around day 10 and day 15, when the fungal community and the larval population increased, which would lead to a larger demand of energy. In addition, at day 30, an increase in the abundance of genes involved in wood degradation was observed again; this concurred with the emergence of the F1 generation from the galleries, raising again the energy requirements due to flight and the unknown time in which the F1 females are in starvation mode after leaving the colonies. Li and collaborators (109) established the mycetangia dynamics in *Xylosandrus* species, finding that new females fill their mycetangia with the fungal symbionts right before dispersal (109). Accordingly, the fungal population increases during this period. This feeding behavior results in the increase of the fat content (76) required for the development of the exoskeleton, reproductive organs, and wing muscles (110) and in the stimulation of juvenile hormone production (78) and other pheromones (111, 112), as well as allowing the female to survive during the starvation period. Simple sugars resulting from the degradation of complex sugars on day 30 in preemergence females would be subsequently degraded in the initial stages of the new colony, as observed in day 5 in foundress females.

Wood is a poor source of nitrogen and nitrogenous compounds such as essential amino acids, cofactors, and vitamins. The latter act as cofactors needed for all kind of enzymatic functions (78). Hence, wood-feeding insects generally need to establish a mutualism with microorganisms to supply them with nitrogen, amino acids, and vitamins (79). Currently, it is believed that bark and ambrosia beetles associate with fungi that supply them with all essential compounds (24), for example, by translocation and concentration of nitrogen and other trace elements from within the wood toward the beetle galleries (80). On the basis of our results, it is possible, however, that in addition to the potential translocation of nutrients by the fungi, atmospheric nitrogen fixation may be present within galleries, most likely by a predominant *Enterobacter* OTU and possibly by other less common bacteria. These bacteria might be the ones observed at the bottom of the *Raffaelea* layers, which might ensure anoxic conditions, which are essential for nitrogen fixation to take place. *Enterobacter* dominates galleries, particularly during the establishment of the colony and might boost nitrogen supply for both fungi and beetles in this nitrogen-poor environment. Putative nitrogen-fixing bacteria have been also isolated from other bark beetles (81–83). If this hypothesis can be confirmed in future studies, it would show that all cases of fungal agriculture in nature (humans, ants, and termites) are dependent on nitrogen-fixing bacteria (7, 8).

Given the nitrogen supply, the core bacterial players are possibly able to produce all the amino acids. They may thus provide methionine to fungi and beetles that they are unable to produce it themselves. All the other amino acids could be synthesized by either the core bacterial or core fungal symbionts. Many of the OTUs that can possibly synthesize essential amino acids increased in abundance during beetle development and were higher within adults and immature offspring than in galleries. This suggests that they are present as gut symbionts and play a nutritional role that increases in importance during development. Furthermore, the core bacteria may supply the beetle diet with several cofactors and vitamins that the core fungi cannot produce (e.g., pantothenate, biotin, and folate).

Quorum sensing is a communication system that enables bacteria to regulate gene expression in response to cell population densities, resulting in phenotypes and physiological responses that allow bacteria to thrive under different conditions. Our analyses suggest that it is possibly present in some of the bacterial symbionts and may induce the yeasts and *Raffaelea* fungi to switch from mycelial to yeast-like growth (Fig. 4). The proximate mechanisms underlying the nutritionally essential induction of yeast-like “ambrosial growth” in ambrosia fungi (40, 41) is one of the major open questions in the ambrosia beetle-fungus mutualism. Other Ophiostomatales fungi are known to switch growth form due to environmental stimuli (84–86), but it is also possible that the OTUs of quorum-sensing bacteria like the *Enterobacter* or the *Stenotrophomonas* may act as triggers (see Fig. S30, S31, and S32 at https://doi.org/10.6084/m9.figshare.12477593). It would be worth isolating these bacteria and testing them in interaction assays with the *Raffaelea* ambrosia fungi.

### Conclusion.

Studies on the ambrosia beetle-fungus symbiosis usually focus on the beetles and their filamentous fungal associates but largely neglect other microbes such as yeasts and bacteria (1, 18, 25). Here we show that yeasts and bacteria are particularly common in young galleries, at the bottom of *Raffaelea* growth, and within the bodies of foundresses and offspring. We also show that these yeasts and bacteria have capabilities in degrading plant polymers, in fixation of atmospheric nitrogen, and in the production of amino acids, cofactors, and vitamins that the filamentous fungi miss. This suggests that yeasts and bacteria have an underappreciated role for ambrosia beetles and their *Raffaelea* fungal mutualists to assist their growth, especially early in gallery development. These findings are corroborated by a recent study that showed species-specific associations between ambrosia fungi and yeasts (including *Candida* and *X. affinis* [32]). Experiments testing the roles of specific yeasts and bacteria can be expected to provide promising results. Overall, it is very likely that the ambrosia beetle-fungus mutualism will soon turn out to be a multipartite symbiosis with additional yeast and bacterial players. This study lists a few candidate microorganisms whose capabilities and interactions with the beetles and the *Raffaelea* ambrosia fungi need to be studied and tested experimentally.

## MATERIALS AND METHODS

### Study species.

Xyleborus affinis Eichhoff is native to the tropical and subtropical Americas and widely distributed in the southeastern United States (87, 88). It was introduced into Africa, Asia, Australia, Europe, and the Pacific Islands, including Hawaii (89, 90). X. affinis is extremely polyphagous using a wide variety of host plants (248 species), including angiosperms as well as gymnosperms (90). Similar to other ambrosia beetles in the subtribe Xyleborini, *X. affinis* displays sib-mating, haplodiploidy, sexual dimorphism, and strongly female-biased sex ratios (90). Fertilized females disperse from their natal nest and bore a branching tunnel system in the xylem of dead or unhealthy hosts. Tunnel walls they inseminate with symbionts they transmit in oral mycetangia or their guts (91). *X. affinis* may inhabit and expand the same gallery for multiple generations over several years and is regarded among the most social beetles (90). They exhibit a cooperatively breeding social system, defined by some adult daughters staying and engaging as temporal workers in the maternal nest, which may cobreed and overtake the nest (14). Quite uniquely also, larvae engage in social hygienic tasks (39). Adult males are flightless, and their only function is to fertilize their sisters (92).

### Medium preparation, beetle, rearing, and sample collection.

Beetle rearing medium was prepared by the method of Biedermann et al. (53), using Persea schiedeana (Chinini) sawdust. Thirty-six adult female foundresses were individually introduced into rearing tubes. Six colonies (tubes) were dissected every 5 days, i.e., at 5, 10, 15, 20, 25, and 30 days after the initiation of the colony. This captures the whole life cycle of *X. affinis* within artificial rearing tubes, from a single mother that needs to feed on the microbial layers covering the gallery walls to her laying eggs (day 5), the development of first larval instars (day 10), second and third larval instars (days 15 and 20), pupae and adult sons (day 25), and adult daughters (day 30) (Fig. 9). Foundresses and offspring of all life stages (larvae, pupae, not fully sclerotized (= teneral) males and females, adult males) were counted, and if available, six individuals were pooled and used for microbiome sequencing. The heads and abdomens of foundresses were processed separately to consider variation between gut and oral mycetangia contents. We focused the study on females, because females are the ones that found new galleries and transmit starter cultures of microbes in oral mycetangia. Therefore, it is particularly interesting what microbes are found in the heads of foundresses immediately after gallery foundation (i.e., day 5) and right before adult female emergence (i.e., day 30). A section of the gallery tunnels was collected for microbiome sequencing at each sampling time to cover the development of the microbiome throughout gallery development and beetle life stages. For an overview of the whole sampling scheme, see Fig. 1.

### DNA extraction library construction and metabarcoding.

DNA extraction was performed following the protocol described by Latorre et al. (93). Sequencing was conducted at FISABIO Service of Sequencing and Bioinformatics (Valencia, Spain). Amplicons of the 16S rDNA V3 region and the 18S rDNA region were generated to determine bacterial and fungal diversity. 18S rDNA region primers were SSUfungiF (5′-TGGAGGGCAAGTCTGGTG-3′) and SSUFungiR (5′-TCGGCATAGTTTATGGTTAAG-3′). DNA libraries were constructed using Nextera XT adapters (Illumina Inc.) and Kappa polymerase (Kappa HiFi Hotstart Ready Mix [catalog no. KK2602; Kappa Biosystems]), and purified using Agencourt Ampure XP magnetic beads (catalog no. A63881; Beckman Coulter). Libraries were pooled to an equimolar concentration and sequenced by MiSeq (reagent kit V3, 600 cycles). The raw data were deposited in NCBI’s SRA archive under BioProject accession number PRJNA561207.

### Sequence assembly and taxonomic annotation.

A total of 8,650,891 paired reads were obtained from 28 high-quality sample libraries for the 16S rRNA marker and 2,691,294 paired reads from 27 high-quality libraries for the 18S rRNA marker (see Table S1 at https://doi.org/10.6084/m9.figshare.12477593). PRINSEQ-lite 0.20.4 (94) was used to trim the 3′ ends of the raw reads and remove positions with a quality score of <20; reads with a mean quality score of ≥20 and a length of ≥50 nucleotides were kept. Paired reads with overlapping ends were joined using the default parameters of the fastq-join tool of the ea-utils package, release 1.1.2-537 (95). Detection and removal of chimeric sequences were done with mothur v.1.25.0 (96), using the Greengenes database (v13_8_99) as the template for the 16S marker (https://greengenes.secondgenome.com/?prefix=downloads/greengenes_database/gg_13_5/); for the 18S rDNA marker, a self-modified version of the QIIME release of the SILVA database v128 was used (97, 98). The modification consisted of clustering 60 new *Raffaelea* sequences extracted from the SILVA repository (https://www.arb-silva.de/search/) together with the 16 *Raffaelea* sequences present in the SILVA database. Clustering was performed using the default parameters of vsearch (v2.3.4) (99, 100) and an identity threshold of 0.99. Consensus sequences obtained from each cluster were added to the SILVA database after removing the original *Raffaelea* sequences.

Operational taxonomic units (OTUs) were picked by open-reference command and defined by clustering at 3% divergence (97% similarity) using the Greengenes database (101) and suppressing the lane mask filter step. The resulting OTU table was converted into a .tsv format with Python’s biom-format package (v. 2.1.5) (102) to filter OTUs from chloroplast, archaeal, mitochondrial, and cyanobacterial sequences and remove those with low abundance (five reads or less per sample). To distinguish each OTU in all analyses performed, a unique identifier (ID) was assigned. This unique identifier was defined by an increasing number followed by the OTU ID and the taxonomic annotation (see Table S4 at https://doi.org/10.6084/m9.figshare.12477593).

### Alpha-diversity analysis and phylogenetic-tree construction.

Rarefactions were produced from the filtered OTUs by running the multiple_rarefactions.py script (QIIME v 1.8.0) with the following parameters: *x* = 1,500, *m* = 10, *s* = 5, and *n* = 10. Diversity indexes (observed OTUs, Shannon, Chao1, and Simpson) were calculated, collated, and plotted with scripts of QIIME (v. 1.8.0) (see Fig. S34 and 35 at https://doi.org/10.6084/m9.figshare.12477593). The alpha-diversity index and OTU richness were plotted by “vegan” and “stats,” and an analysis of variance (ANOVA) and Tukey test were performed to detect significant differences between the diversity indexes across days, body parts, and sample types using R (v.3.3.1).

The visualizations of the bacterial and fungal microbiomes were conducted with the software Graphical Phylogenetic Analysis (GraPhlAn) (103). The relative taxonomic abundances of the samples were displayed with collapsed histograms plotted by “RColorBrewer” and “ggplot2” libraries in R (v.3.3.1). Phylogenetic trees of bacterial and fungal OTUs were constructed using FastTree 2.1.3. (104), by using the script make_phylogeny.py of QIIME 1 (100). The phylogenetic trees were pruned, removing all the OTUs with a relative frequency of <0.1% by the “filter_tree.py” script of Qiime1. The resulting trees were transformed to dendrograms using “ape v5.3” in R (v.3.3.1). To visualize the relative abundances of the most frequent OTUs, we constructed a heatmap for every sample type. We displayed the OTUs using the scripts “ape,” “vegan,” and “RColorBrewer” in R (v.3.3.1).

### Visualization of galleries through microscopy.

Samples from the gallery walls were collected for visualization by scanning electron microscopy (SEM) from three galleries, each at days 3, 5, 10, 15, 20, 25, and 30 and for light microscopy (LM) from three galleries, each at days 5, 10, 15, 20, 25, and 30. SEM was done on a FEI Quanta 250 FEG microscope after samples were prepared following the protocol described by Hermida-Montero et al. (105). Light microscopy was done on a DMI 6000B Leica inverted microscope and prepared following the protocol of Guillen et al. (106). Samples from the gallery walls were collected for visualization by scanning electron microscopy (SEM) from 3 galleries each at an age of 3, 5, 10, 15, 20, 25 and 30 days and for light microscopy from 3 galleries each at an age of 5, 10, 15, 20, 25 and 30 days. SEM was done on a FEI Quanta 250 FEG microscope after samples were prepared following the protocol described in Hermida-Montero and collaborators (105). Samples for light microscopy (LM) was done on DMI 6000B LEICA Inverted Microscope were prepared following the protocol of Guillén and collaborators (106).

### Functional metabolic prediction of the bacterial and fungal OTUs.

Functional annotations of the bacterial microbiome were conducted with Phylogenetic Investigation of Communities by Reconstruction of Unobserved States (PICRUSt) (version 1.1.3) (107), which allows us to predict bacterial metabolic functions based on 16S rRNA sequences using the Greengenes database of reference genomes. The enrichment analysis of pathways was performed based on the Kyoto Encyclopedia of Genes and Genomes (KEGG) database. We performed the PICRUSt normalization, which is selective for OTUs within the Greengenes database, thus inferring the metabolic profile of 267 bacterial OTUs (with a median of 60,837 reads per sample). This means that a putative metabolic profile could be assigned to about 60% of the bacterial OTUs in our samples.

To illustrate dissimilarities based on KEGG Orthologies (KOs) and L3 KEGG level categories, nonmetric multidimensional scaling (NMDS) across the samples was carried out using the Bray-Curtis distance matrix. The effects of the factors sample type and sampling time on KEGG functional categories were evaluated with a permutational multivariate analysis of variance (PERMANOVA) using a Bray-Curtis dissimilarity matrix that was previously calculated considering the relative abundances of functional categories in all samples. The significance threshold for the PERMANOVA was set at *P* < 0.001. When the PERMANOVA was significant, differences between samples were determined with multiple pairwise comparisons using a Wilcoxon test with Bonferroni correction set at *P* < 0.01. All the analyses were performed using “vegan” and “mass” in R (v.3.3.1). To visualize shared and private KOs between adults, offspring of different life stages, and galleries across development (days 5, 10, 15, 20, 25, and 30), we constructed an UpSet plot using the R package UpsetR (108).

To gain insights into the metabolic potential of the fungi associated with the ambrosia beetles, available genomes of the phylogenetically most closely related fungi (*n* = 33 genomes; see Table S5 at https://doi.org/10.6084/m9.figshare.12477593) were selected, and the core genome and their metabolic profile were determined following the methodology reported by Ibarra-Juarez et al. (46). Additionally, the presence of genes and specific metabolic pathways (degradation of plant and fungal cell wall components; biosynthesis of essential amino acids, cofactors and vitamins; nitrogen fixation; quorum sensing and biofilm production) were analyzed and plotted in a heatmap using “ggplot2” and “vegan” for R (v.3.3.1). The main components of the plant cell wall are cellulose, hemicellulose (1,4-β-d-xylan), pectin [poly(1,4-α-d-galacturonide)], lignin (3,4-dihydroxybenzoate), and the simple-sugar components of pectin and hemicellulose, arabinose, and rhamnose. The fungal cell wall consists of chitin (*N*-acetylglucosamine), glucan (1,3-β-glucan), and mannan (1,4-β-mannan). The metabolic pathways for the degradation of these components are given in Fig. S36 at https://doi.org/10.6084/m9.figshare.12477593.

To infer the metabolic capability of the bacterial microbiome in specific pathways, the relative frequencies of all genes involved in these pathways were calculated per sample. A boxplot for the sampling times (5, 10, 15, 20, 25, and 30 days) and the type of sample was generated using “ggplot2” and “vegan” in R (v.3.3.1). To analyze the bacterial contribution to the different steps of the selected pathways, we ran the script metagenome_contributions.py of PICRUSt (107). This script generates the relative frequency of the KO present in the sample by a specific OTU. We built a stack histogram using the sum of the averages of these relative frequencies of all KOs involved in a metabolic pathway from a specific OTU.

To analyze the capability of the microbiome to produce biofilm, we calculated the relative frequencies per sample of the genes involved in biofilm production and the genes involved in planktonic stage, based on the KOs predicted by PICRUSt. We consider the KOs K00688, K00694, K00703, K00975, K01991, K03087, K03566, K04333, K04334, K04335, K04336, K04761, K06204, K07173, K07638, K07659, K07676, K07677, K07678, K07687, K07689, K07781, K07782, K11531, K11931, K11935, K11936, K11937, and K14051 to be involved in biofilm production and the KOs K02398, K02402, K02403, K02405, K02425, K02777, K03563, K03567, K05851, K07648, K07773, and K10914 to be involved in planktonic stage.

### Data availability.

The raw data were deposited in NCBI’s SRA archive under BioProject accession number PRJNA561207.
