# Supplementary figures and images for: Evidence for Succession and Putative Metabolic Roles of Fungi and Bacteria in the Farming Mutualism of the Ambrosia Beetle Xyleborus affinis
Source: mSystems. 2020 Sep 15;5(5):e00541-20. doi: 10.1128/mSystems.00541-20 (PMC7498683; doi:10.1128/mSystems.00541-20)

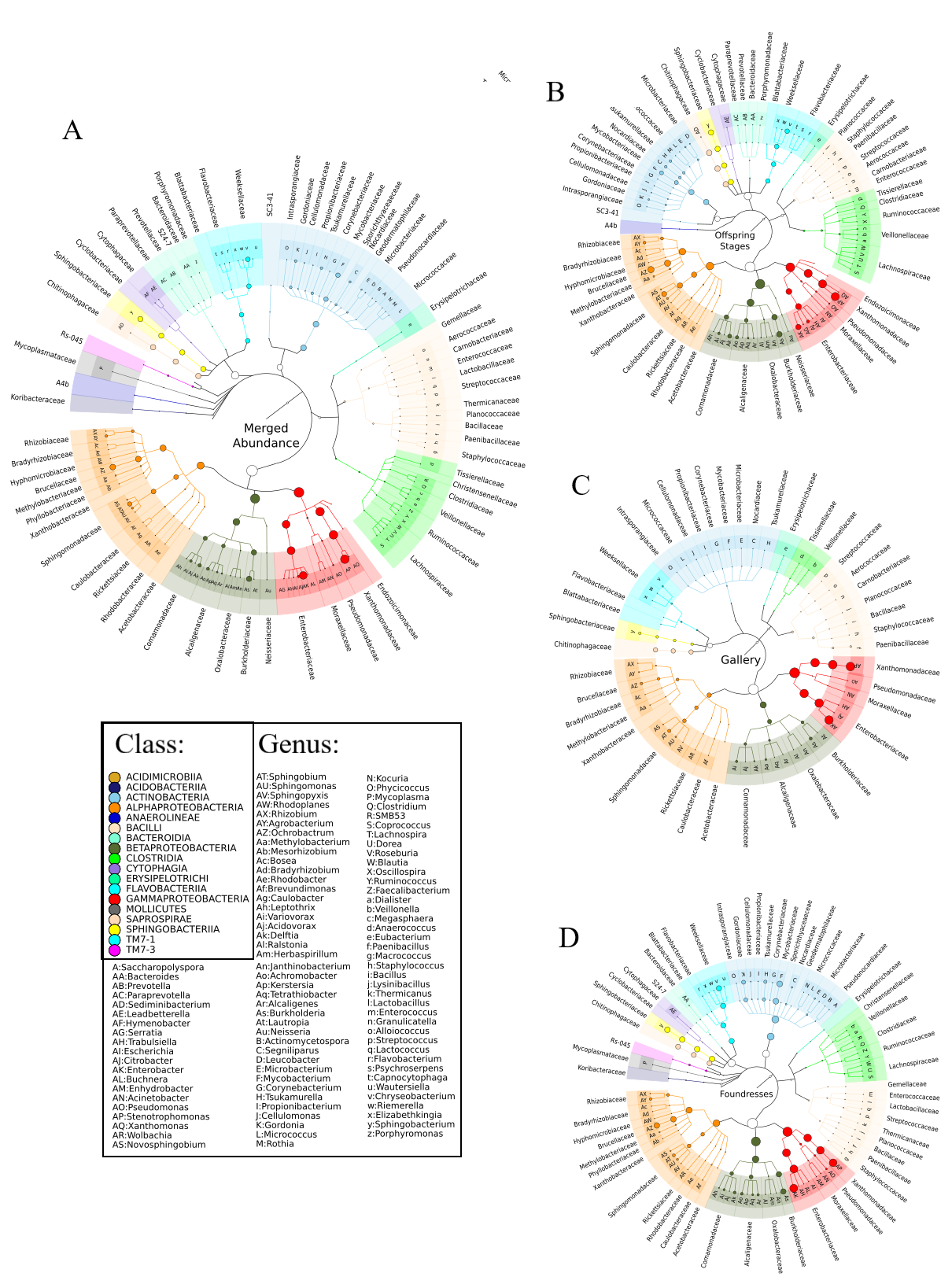

Supplement: FIG S1 [file mSystems.00541-20-sf001.tif]

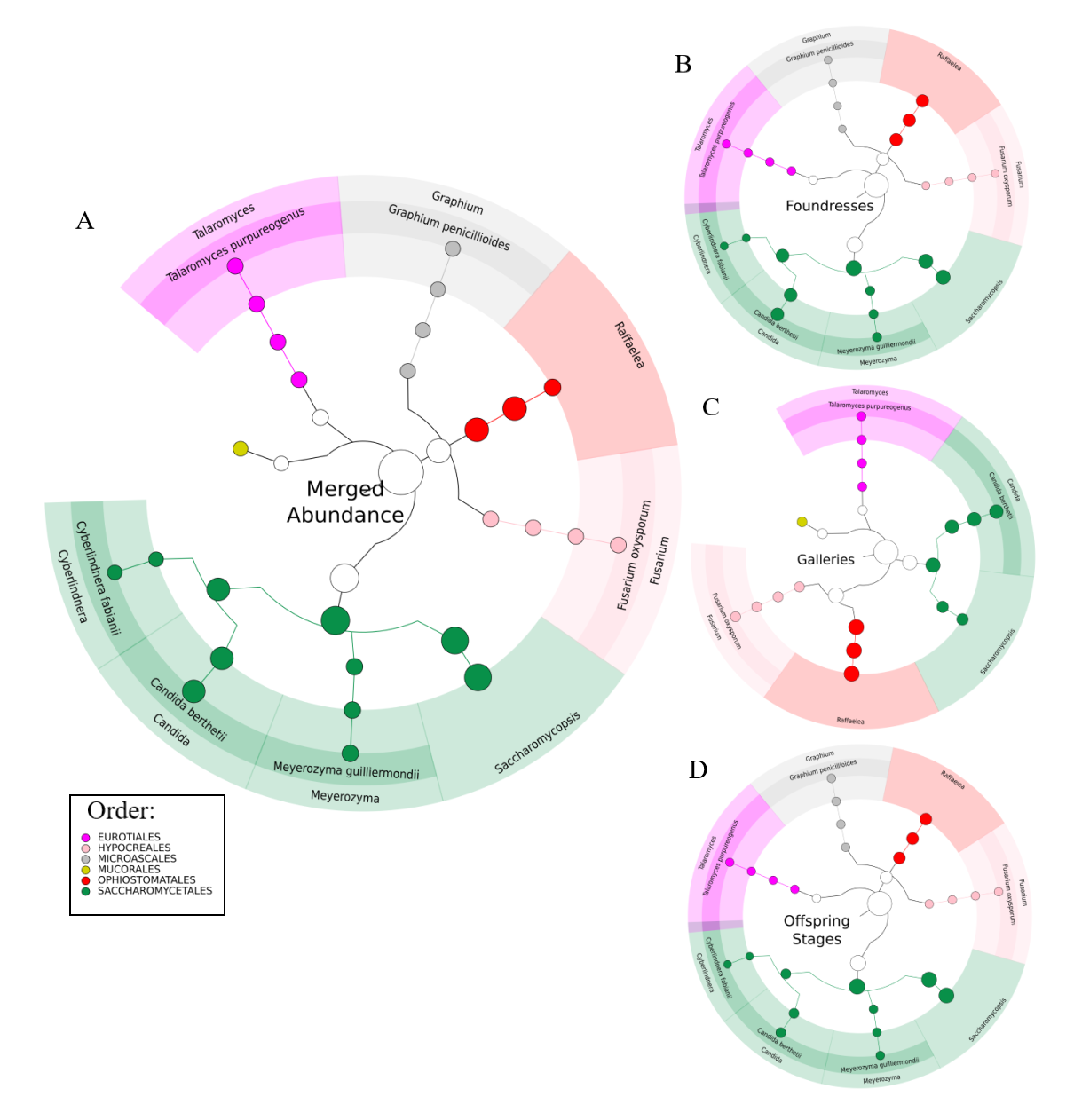

Supplement: FIG S2 [file mSystems.00541-20-sf002.tif]
